# CEACAM6 as a machine learning derived immune biomarker for predicting neoadjuvant chemotherapy response in HR+/HER2− breast cancer

**DOI:** 10.3389/fimmu.2025.1662004

**Published:** 2025-08-27

**Authors:** Dalang Fang, Jie Lin, Jin Wang, Qingxiao Nong, Shouwen Tao, Bimin Lu, Yanrong Yu, Hao Peng, Yingying Tian, Qunying Su, Yanfei Ma, Yuanlu Huang

**Affiliations:** ^1^ Department of Gland Surgery, Affiliated Hospital of Youjiang Medical University for Nationalities, Key Laboratory of Tumor Molecular Pathology of Baise, Baise, Guangxi, China; ^2^ Department of Pathology, Affiliated Hospital of Youjiang Medical University for Nationalities, Baise, Guangxi, China; ^3^ Department of Gland Surgery, Baise People’s Hospital, Baise, Guangxi, China

**Keywords:** machine learning, immune infiltration, HR+/HER2-breast cancer, CEACAM6, neoadjuvant chemotherapy (NAC)

## Abstract

**Background:**

Hormone receptor-positive/human epidermal growth factor receptor 2-negative (HR+/HER2−) breast cancer is the most common subtype, characterized by heterogeneous neoadjuvant chemotherapy (NAC) responses and low pCR rates. Existing biomarkers have limited predictive accuracy, hindering personalized treatment. This study aimed to identify predictive biomarkers for NAC response and explore their therapeutic potential in HR+/HER2− breast cancer.

**Methods:**

We integrated 497 HR+/HER2− samples from TCGA and 956 from nine GEO datasets (training set: n=708; test set: n=248). Differentially expressed genes (DEGs) between tumors and normal tissues (TCGA) and between pCR and residual disease (RD) groups (GEO) were identified. Overlapping DEGs were further screened using LASSO, random forest, and SVM-RFE algorithms. Predictive models were constructed with 10 machine learning algorithms and interpreted using SHAP. Gene set enrichment analysis (GSEA), CIBERSORT-based immune infiltration, and drug sensitivity prediction using oncoPredict and GDSC2 were performed. Immunohistochemistry (IHC) was conducted on paired pre/post-NAC samples (n=9). Clinical correlation was analyzed in a retrospective cohort of 106 HR+/HER2− NAC patients.

**Results:**

Thirty-eight overlapping DEGs were identified, and four key genes (CEACAM6, MELK, RARRES1, BIRC5) were selected. NeuralNet showed the best model performance (AUC=0.816). CEACAM6 was the top-ranked SHAP feature, with high expression predicting RD and was associated with poor survival (p=0.014). GSEA revealed CEACAM6-high tumors were enriched in drug resistance pathways (such as oxidative phosphorylation), while low expression correlated with immune activation. Immune analysis showed pCR tumors had more effector cells (Tfh, γδ T cells, M1 macrophages), whereas RD tumors were enriched in Tregs and resting mast cells. CEACAM6 positively correlated with Tregs and naïve CD4+ T cells, and negatively with CD8+ T cells and M1 macrophages. CEACAM6-high tumors had higher IC50 for six NAC-related drugs. IHC confirmed persistent CEACAM6 expression in RD tumors post-NAC. Clinically, pCR patients had higher lymphocyte counts and more frequent N2–N3 nodal status.

**Conclusion:**

CEACAM6 is a promising predictive biomarker in HR+/HER2− breast cancer, associated with chemoresistance and immune suppression. Machine learning models integrating immune signatures and pathway features may optimize personalized NAC strategies.

## 1 Introduction

Breast cancer is one of the most common malignant tumors among women worldwide, with over 2.3 million new cases and nearly 680,000 deaths reported globally in 2024, posing a serious threat to women’s health and survival (1). Hormone receptor-positive/human epidermal growth factor receptor 2-negative (HR+/HER2−) breast cancer accounts for approximately 60-70% of all breast cancer cases, making it the most prevalent subtype in clinical practice (2). Despite the established role of NAC in breast cancer management, the therapeutic benefit remains markedly heterogeneous in patients with the HR+/HER2− subtype. Compared with triple-negative breast cancer (TNBC) and HER2-positive subtypes, HR+/HER2− tumors have significantly lower pathological complete response (pCR) rates, generally only 10-15% (3). Numerous studies have demonstrated that this subtype responds poorly to cytotoxic chemotherapy. A subset of patients exhibit substantial residual disease (RD) after standard NAC, leading to higher rates of long-term recurrence and significantly compromising prognosis (4, 5). However, existing predictive tools such as Ki-67, TILs, and gene expression signatures suffer from several limitations in HR+/HER2− breast cancer (6). The predictive performance of Ki-67 is inconsistent due to variability in staining protocols, scoring systems, and cut-off thresholds across laboratories, leading to poor reproducibility and limited clinical utility. Similarly, although TILs have been recognized as predictive biomarkers in triple-negative and HER2-positive subtypes, their relevance in HR+/HER2− tumors remains unclear, as these tumors often exhibit lower immune cell infiltration and reduced immune activation. Multigene signatures (such as Oncotype DX, MammaPrint) are primarily designed for prognostication and guiding adjuvant endocrine therapy decisions, rather than specifically predicting chemotherapy benefit in the neoadjuvant setting (7). Moreover, the HR+/HER2− subtype is characterized by an immunologically “cold” microenvironment, with low CD8+ T cell infiltration, high expression of immune suppressive cells (such as Tregs), and reduced immunogenicity. These unique features contribute to poor responsiveness to cytotoxic chemotherapy and highlight the urgent need for novel biomarkers that can more accurately predict NAC response in this subtype.

With the advancement of omics technologies, researchers have begun to explore transcriptomic features, tumor immune microenvironment status, and peripheral immune markers for their potential value in predicting chemotherapy response. For example, transcriptome studies based on RNA sequencing have identified differentially expressed genes (DEGs) and constructed predictive models from datasets such as TCGA and GEO, revealing that the expression of certain genes involved in cell cycle and DNA damage repair correlates with pCR (8), highlighting the importance of intrinsic tumor molecular states in determining chemosensitivity. In addition, studies have reported that patients with high tumor-infiltrating lymphocyte (TIL) levels are more likely to achieve pCR (9, 10), and that peripheral lymphocyte counts are also associated with treatment response (11). However, these immune-related biomarkers still face significant limitations, including variability in detection methods and poor reproducibility across clinical settings. Compared to triple-negative and HER2-positive breast cancer, HR+/HER2− breast cancer displays a distinct immunosuppressive microenvironment, characterized by lower infiltration of effector immune cells such as CD8+ T cells and natural killer (NK) cells, and a higher abundance of immunosuppressive populations, including regulatory T cells (Tregs) and myeloid-derived suppressor cells (MDSCs) (12). Tregs suppress effector T cell function by secreting inhibitory cytokines such as TGF-β and IL-10, while MDSCs impair T cell activation, jointly contributing to chemoresistance and the relatively low pCR rates (10–15%) observed in this subtype (13, 14). In this study, we investigate a key biomarker associated with response to neoadjuvant chemotherapy (NAC) in HR+/HER2− breast cancer, incorporating CIBERSORT-based immune profiling to explore its potential role in shaping the immunosuppressive landscape and mediating resistance to chemotherapy.

In recent years, artificial intelligence (AI), particularly machine learning (ML) approaches, has shown remarkable advantages in biomarker discovery for cancer (15). Unlike traditional univariate statistical methods, ML algorithms can integrate high-dimensional, multimodal data and automatically identify complex, non-linear combinations of variables with predictive value. Algorithms such as least absolute shrinkage and selection operator (LASSO) regression, random forest (RF), and support vector machine-recursive feature elimination (SVM-RFE) have demonstrated excellent performance in high-throughput dimensionality reduction and feature selection. Several studies have applied these algorithms to construct prediction models for NAC response in breast cancer (16, 17); however, most were limited by small sample sizes, lack of cross-validation, and poor model generalizability, limiting their clinical applicability. Moreover, few existing studies have integrated ML-based models with immune infiltration analysis, biological pathway enrichment, and clinicopathologic variables-limiting their biological interpretability and failing to support truly individualized therapeutic decision-making (18). In terms of drug resistance mechanisms, previous work has mostly focused on pathway-level analysis, lacking systematic interpretations from the perspective of driver genes and their interactions with immune evasion or drug response (19, 20). In HR+/HER2− breast cancer, chemotherapy resistance may be synergistically driven by immunosuppressive mechanisms such as Treg recruitment and effector T cell dysfunction, but basic and clinical research exploring these mechanisms remains relatively limited (9). Therefore, HR+/HER2− breast cancer continues to face significant challenges in the NAC setting, including pronounced heterogeneity in treatment efficacy, low pCR rates, and the lack of efficient and widely applicable predictive tools. Although some studies have attempted to identify predictive biomarkers, they are frequently constrained by limited sample sizes, single data sources, inadequate external validation, and lack of multimodal integration-making them insufficient for clinical use. Additionally, there remains a lack of predictive models that comprehensively integrate AI-based algorithms with immune context, molecular pathways, and drug response information, which restricts their translational utility and biological interpretability.

This study aimed to identify key molecular biomarkers predictive of NAC sensitivity in HR+/HER2− breast cancer and to explore their potential as therapeutic targets. We integrated data from TCGA and nine publicly available GEO datasets, encompassing a total of 1,453 HR+/HER2− breast cancer samples, and applied multiple machine learning algorithms to construct predictive models. These models were validated in independent cohorts to assess their predictive performance. To enhance model interpretability, we incorporated SHAP (SHapley Additive exPlanations) analysis and conducted immune infiltration analysis via CIBERSORT, pathway enrichment using GSEA, and drug sensitivity prediction through the GDSC database to comprehensively evaluate the relationship between key genes, the immune microenvironment, and chemotherapeutic responsiveness. In addition, we retrospectively analyzed a clinical cohort of 106 HR+/HER2− breast cancer patients receiving NAC and performed paired immunohistochemical (IHC) validation of CEACAM6 expression before and after treatment to examine its correlation with pCR status. These efforts provide translational evidence to support CEACAM6 as a predictive biomarker and potential therapeutic target in the NAC setting for HR+/HER2− breast cancer.

## 2 Materials and methods

### 2.1 Data acquisition, batch correction, and integration

In this study, nine transcriptomic datasets related to neoadjuvant chemotherapy (NAC) in breast cancer were systematically retrieved from the GEO database (https://www.ncbi.nlm.nih.gov/geo/). Inclusion criteria were: (1) pre-treatment biopsy transcriptomic data from patients receiving anthracycline- and taxane-based NAC (≥4 cycles), (2) availability of clinical response data (pCR or RD), and (3) HR+/HER2− subtype confirmed by immunohistochemistry or gene expression. Datasets lacking clinical response, HR+/HER2− subtype, or using non-standard regimens were excluded. Ultimately, 956 HR+/HER2− samples from nine datasets (GSE20194, GSE20271, GSE25066, GSE41998, GSE163882, GSE34138, GSE32646, GSE22093, GSE23988) were included. Among them, five datasets were used for training (GSE20194, GSE20271, GSE25066, GSE41998, GSE163882), and four for testing (GSE34138, GSE32646, GSE22093, GSE23988). Detailed dataset characteristics are summarized in **Table 1**.

**Table 1:** Summary of transcriptomic datasets and sample distribution in the training, test, and TCGA cohorts.

Cohort	Dataset (all sample)	HR+/HER2- breast cancer	
		RD	pCR
Train	GSE20194 (n=278)	n=133	n=7
	GSE20271 (n=178)	n=87	n=6
	GSE25066 (n=508)	n=259	n=36
	GSE41998 (n=279)	n=99	n=12
	GSE163882 (n=222)	n=56	n=13
	Summary	634 (89.54%)	74 (10.45%)
Test	GSE34138 (n=178)	n=109	n=10
	GSE32646 (n=115)	n=50	n=5
	GSE22093 (n=103)	n=32	n=10
	GSE23988 (n=61)	n=25	n=7
	Summary	216 (87.10%)	32 (12.90%)
TCGA	BRCA (n=1226)	Normal=113	HR+/HER2- =497

Batch effects were independently corrected for training and test cohorts using the ComBat function from the sva package in R software (version 4.5.0). Principal component analysis (PCA) was conducted to evaluate the consistency and comparability of gene expression profiles after batch correction.

Additionally, RNA-seq and clinical outcome data of the TCGA-BRCA cohort were obtained from The Cancer Genome Atlas (TCGA, https://portal.gdc.cancer.gov/). Normal breast tissues and HR+/HER2- tumor samples were extracted for differential expression and prognostic analyses.

### 2.2 Identification of differentially expressed genes

Differential expression analysis was first performed between tumor and normal tissues in HR+/HER2- breast cancer samples from the TCGA cohort (113 normal *vs*. 497 tumor). To reduce background noise and prioritize tumor-intrinsic candidates, only genes differentially expressed in both tumor *vs*. normal and pCR *vs*. RD comparisons were retained for downstream analysis. Following data preprocessing and normalization, CPM values were calculated using the edgeR package, and low-expression genes were filtered. Linear modeling was conducted using the limma package (21), and empirical Bayes moderation was applied to identify DEGs. Genes with |log2 fold change| > 1 and false discovery rate (FDR) < 0.05 were considered significant, as these thresholds are commonly used in transcriptomic studies to balance sensitivity and specificity while capturing biologically relevant genes.

Subsequently, within the GEO training cohort, differential expression analysis was performed between the pathologic complete response (pCR) and residual disease (RD) groups. The same preprocessing and statistical approach were used to identify DEGs between these two subgroups. Volcano plots and heatmaps were generated using the ggplot2 package to visualize the results.

### 2.3 Feature selection of overlapping DEGs via machine learning

Genes overlapping in both DEG sets from the TCGA tumor *vs*. normal comparison and the GEO pCR *vs*. RD comparison were defined as candidate genes. To identify key features from this gene set, three machine learning methods were applied. First, LASSO logistic regression was performed using the glmnet package with 10-fold cross-validation to select genes with non-zero coefficients. Second, the Random Forest (RF) algorithm was implemented using the randomForest package (22), and genes with a MeanDecreaseGini > 3.5 were retained. Third, Support Vector Machine-Recursive Feature Elimination (SVM-RFE) was conducted using the e1071 package, with the optimal gene subset determined based on the minimal cross-validation error. Genes identified by each method were used for subsequent modeling and downstream analyses. To enhance the robustness of feature selection and minimize method-specific bias, we prioritized genes consistently identified by all three algorithms, thereby highlighting the most reliable predictors.

### 2.4 Model construction and feature interpretability

Intersection genes identified from LASSO, RF, and SVM-RFE were considered final feature genes. Using their expression profiles as predictors, diagnostic models were trained on the GEO training cohort and validated on the GEO test cohort. We applied 5-repeated 5-fold cross-validation using the caret package to train and evaluate ten commonly used machine learning algorithms: Partial Least Squares (PLS), Random Forest (RF), Decision Tree (DTS), Support Vector Machine with radial kernel (SVM), Logistic Regression (Logistic), k-Nearest Neighbors (KNN), Extreme Gradient Boosting (XGBoost), Gradient Boosting Machine (GBM), Neural Network (NeuralNet), and Generalized Linear Model with Boosting (glmBoost). ROC curves and AUC values were calculated based on the test cohort to assess model performance, and the algorithm with the highest AUC was selected as the optimal classifier. Model interpretation was then conducted using DALEX (https://jmlr.org/papers/v19/18-416.html) and kernelshap (https://arxiv.org/abs/1705.07874) to compute SHAP values, and gene contribution was visualized through bar plots, bee swarm plots, and individual-level waterfall charts. To enhance model interpretability, we applied SHAP (SHapley Additive exPlanations) analysis using the kernelshap and shapviz R packages. The SHAP values were estimated using the permshap() function with a permutation-based approach. This method was chosen to improve computational efficiency and reduce variance, especially in our high-dimensional dataset. For interpretability, we focused on the final feature genes, which were consistently identified by LASSO, Random Forest, and SVM-RFE algorithms.

We generated summary bar plots, bee swarm plots, and waterfall plots to visualize both global and sample-level feature importance. While SHAP provides an intuitive explanation of model predictions, we acknowledge several limitations. First, SHAP assumes feature independence, which may not fully apply in gene expression data due to gene-gene correlation. Second, in small-sample settings, SHAP values may be sensitive to input variation, potentially affecting stability. Third, SHAP captures associations but does not imply causation. Therefore, we used SHAP primarily for descriptive insight rather than model selection. We recommend interpreting the results in combination with biological knowledge and other analytical findings.

### 2.5 Expression and prognostic value of key genes

In the TCGA-BRCA dataset, boxplots were used to compare the expression of key genes between normal and HR+/HER2- tumor tissues. Expression distributions in RD and pCR subgroups were also evaluated using boxplots in both GEO training and test cohorts. Prognostic relevance was assessed by Kaplan-Meier overall survival (OS) analysis, stratifying TCGA samples into high and low expression groups based on the feature gene expression. Genes with significant survival associations were designated for further analysis.

### 2.6 Functional enrichment analysis of key genes

To explore the biological mechanisms underlying key genes, gene set enrichment analysis (GSEA) was conducted using GEO training cohort data. For each gene, samples were divided into high and low expression groups. LogFC values were calculated between groups, and genes were ranked accordingly. KEGG was selected as the reference pathway database because GSEA focuses on coordinated expression shifts across entire gene sets, which is more suitable for evaluating transcriptional activity and pathway-level immune phenotypes than categorical enrichment provided by GO or BP annotation.

GSEA was performed using the clusterProfiler package (23) with the c2.cp.kegg.v7.5.1.symbols.gmt gene set from MSigDB (https://www.gsea-msigdb.org/gsea/msigdb). P < 0.05 was used as the cutoff for significant enrichment. GSEA enrichment plots of the top 5 upregulated and downregulated pathways were generated for each gene to visualize their functional roles.

### 2.7 Immune cell infiltration and immune correlation of key genes

CIBERSORT was applied to estimate the immune cell composition in GEO training samples using the LM22 signature and 1,000 permutations (24). CIBERSORT applies support vector regression to deconvolute bulk gene expression profiles into relative fractions of 22 immune cell types. This method has been extensively used in studies across various cancer types, including breast cancer, due to its robust performance in samples with mixed cell populations and varying tumor purity. Boxplots were generated to compare immune cell fractions between RD and pCR groups using ggplot2 and ggpubr.

To assess the immunological relevance of key genes, their expression data were integrated with immune cell infiltration results. Spearman correlation analysis was used to quantify associations between gene expression and immune cell abundance. linkET was employed to construct coupled correlation networks between immune cells and key genes. Coupled correlation maps were generated to visualize the strength and direction of these associations through edge thickness and color.

### 2.8 Drug sensitivity analysis

To evaluate the potential association between key genes and drug responses to chemotherapeutic and endocrine agents commonly used in HR+/HER2- breast cancer, the oncoPredict package (https://github.com/HuangLabUMN/oncoPredict) was employed. Drug sensitivity prediction was performed using gene expression profiles from the GEO training cohort. GDSC2-derived drug response profiles (GDSC2_Res) and gene expression data (GDSC2_Expr) were used as training datasets (https://www.cancerrxgene.org). GEO expression data were preprocessed, including filtering low-expression genes, normalization, and batch correction. IC50 values were predicted using the calcPhenotype function. Samples were stratified into high and low expression groups based on the median expression of each key gene. Differences in predicted IC50 values for various drugs were compared between groups using the Wilcoxon rank-sum test.

### 2.9 Immunohistochemical protocol for key gene detection in pCR, RD, and normal tissue

Formalin-fixed, paraffin-embedded (FFPE) breast tissue samples were classified into three groups: (1) adjacent non-tumor tissues obtained from radical mastectomy specimens of HR+/HER2− breast cancer patients who had not received neoadjuvant chemotherapy (n = 3); (2) tumor tissues collected via core needle or vacuum-assisted biopsy prior to chemotherapy from patients who subsequently received neoadjuvant chemotherapy (n = 3, RD group; n = 3, pCR group); and (3) matched post-chemotherapy tumor tissues obtained from radical mastectomy specimens of the same patients after six cycles of treatment. A total of 9 FFPE tissue specimens were obtained from the Department of Pathology of our hospital. Consecutive 2.5-μm sections were cut and mounted on poly-L-lysine-coated slides. After baking, the sections were deparaffinized in xylene, rehydrated through graded ethanol, and subjected to antigen retrieval using EDTA buffer (pH 9.0) in a pressure cooker at 500W for 20 minutes, followed by natural cooling and washing in distilled water. Endogenous peroxidase activity was blocked. Sections were then incubated with a CEACAM6-specific primary antibody (1:1000 dilution; Baida Medical Technology Co., Ltd., Suzhou, China) at 37°C for 60 minutes. After PBS washes (3 × 3 minutes), the sections were incubated with MaxVision™ HRP-conjugated secondary antibody (mouse/rabbit) at room temperature for 15 minutes. Following additional PBS washes, immunoreactivity was visualized using freshly prepared DAB substrate under microscopic observation. The reaction was stopped with running water, followed by hematoxylin counterstaining, acid alcohol differentiation (2–3 seconds), bluing, dehydration in graded ethanol, air-drying, and mounting with neutral resin.

### 2.10 Collected an external independent cohort to analyze clinical and pathological features associated with pCR in HR+/HER2- breast cancer

This study included 106 patients diagnosed with HR+/HER2- breast cancer at The Affiliated Hospital of Youjiang Medical University for Nationalities between January 01, 2021, and December 31, 2024. Patients were eligible if they had histologically confirmed invasive breast cancer and underwent NAC. All patients received NAC regimens based on fluorouracil, anthracyclines, and taxanes. Exclusion criteria included prior malignancy, incomplete clinical data, or loss to follow-up. The cohort was stratified into two groups based on NAC response: pathological complete response (pCR, n = 10) and residual disease (RD, n = 96). pCR was defined as the absence of invasive carcinoma in both the breast and axillary lymph nodes, while RD was defined as the presence of residual invasive cancer in the breast and/or axillary lymph nodes. Baseline clinical and pathological characteristics were retrospectively collected from medical records, including age, body mass index (BMI), menopausal status, TNM stage, tumor size, estrogen receptor (ER) and progesterone receptor (PR) expression, Ki-67 index, histological grade, and metastasis status. Blood routine parameters, including neutrophil count, lymphocyte count, and neutrophil-to-lymphocyte ratio (NLR), were obtained prior to NAC. Menopausal status was classified as premenopausal (menstrual cycles ongoing or ceased <12 months) or postmenopausal (no menses for ≥12 months or surgical menopause). ER and PR positivity were defined as ≥10% stained cells, and the Ki-67 index was categorized as <30% or ≥30%.

### 2.11 Statistical analysis

All statistical analyses were conducted using R software (version 4.5.0). Differential expression was assessed using the edgeR and limma packages with |log_2_ fold change| > 1 and FDR < 0.05 as cutoffs. Candidate genes were selected using LASSO logistic regression (glmnet), random forest (randomForest), and SVM-RFE (e1071). Machine learning models were built with the caret package using 5-repeated 5-fold cross-validation across ten algorithms, and model performance was evaluated using ROC curves and AUC values. The best-performing model was interpreted using DALEX and kernelshap to calculate SHAP values. Immune cell proportions were estimated with CIBERSORT, and group differences were tested using the Wilcoxon rank-sum test. GSEA was performed via clusterProfiler, and drug sensitivity was predicted using oncoPredict based on GDSC2 data. Continuous variables were expressed as mean ± standard deviation (SD), and categorical variables as numbers and percentages. Differences between pCR and RD groups were assessed using independent t-tests for continuous variables and Fisher’s exact test for categorical variables, applied when expected cell frequencies were <5 to ensure robust statistical inference. P < 0.05 was considered statistically significant.

## 3 Results

### 3.1 Data integration and cohort characteristics

**Figure 1:**
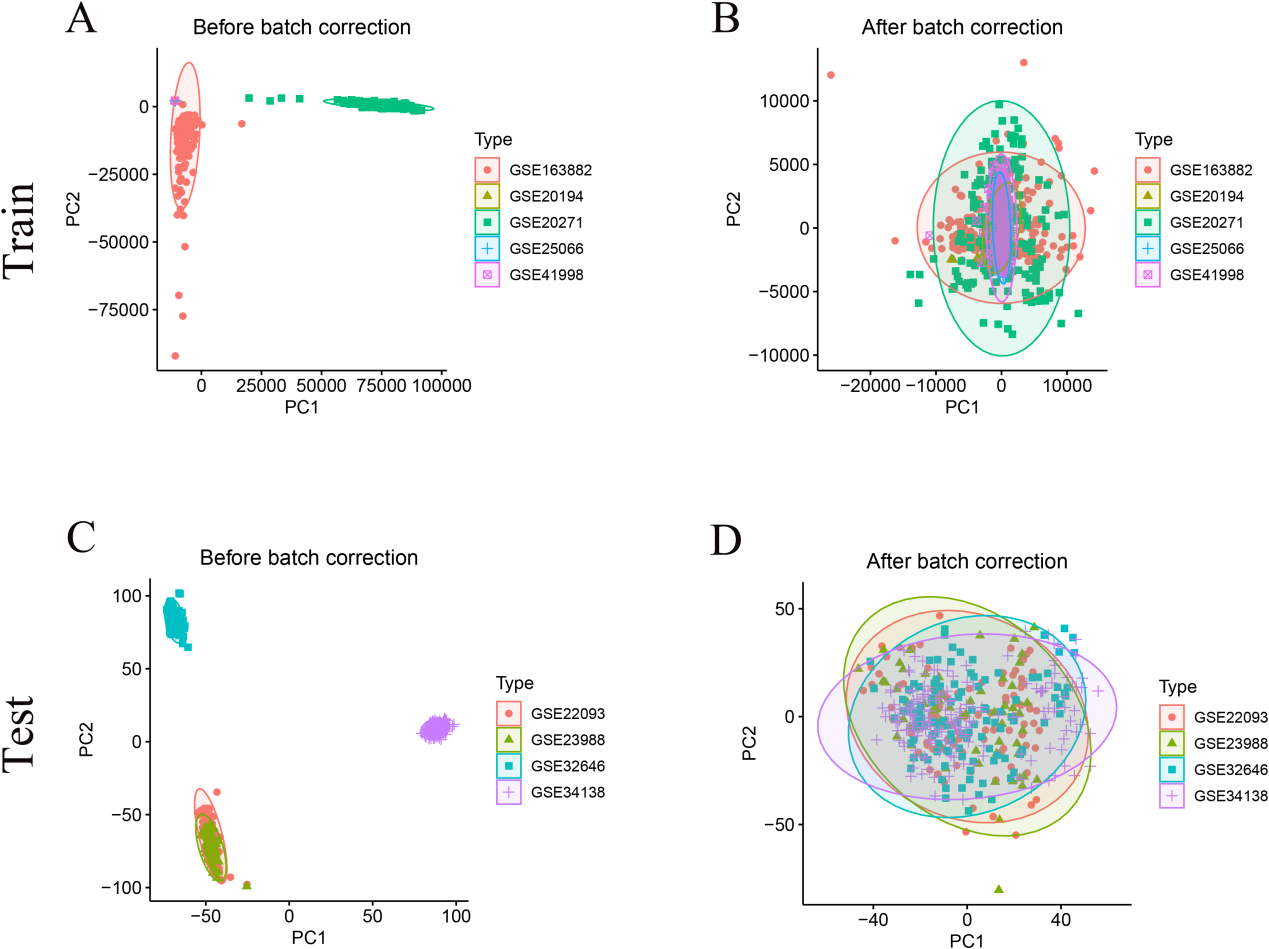
Data integration and batch−effect correction. **(A)** PCA of uncorrected GEO Train cohort shows distinct dataset clustering. **(B)** PCA of the training cohort after ComBat correction demonstrates clustering overlap, indicating removal of batch effects. **(C)** PCA of uncorrected GEO Test cohort shows distinct dataset clustering, which is largely abrogated after correction **(D)**.

A total of 956 HR+/HER2- breast cancer samples from nine GEO datasets were included for model construction and validation (**Table 1**). After batch correction using the ComBat algorithm, PCA analysis demonstrated improved consistency across datasets within the training and test cohorts (**Figures 1A–D**). The training cohort included 708 samples (pCR = 74, RD = 634), while the test cohort included 248 samples (pCR = 32, RD = 216). Additionally, 113 normal and 497 HR+/HER2- tumor tissue samples from the TCGA-BRCA cohort were analyzed to identify differentially expressed genes between normal and tumor tissues.

### 3.2 Identification of differentially expressed genes

**Figure 2:**
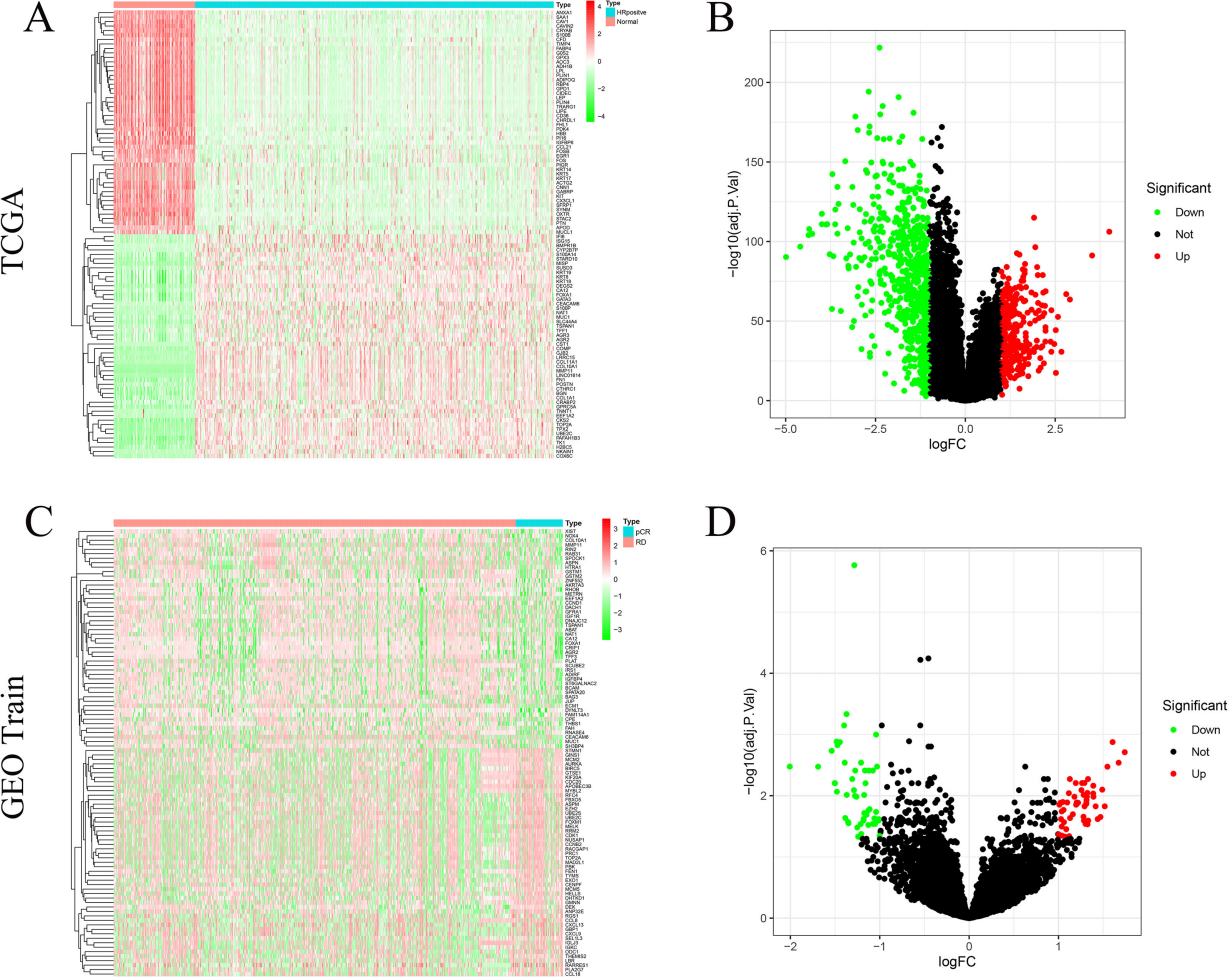
Differentially expressed genes (DEGs). **(A)** Heat−map of the top 100 TCGA DEGs. **(B)** Volcano plot of TCGA HR+/HER2– tumours versus normal breast tissue (678 down− and 306 up−regulated DEGs). **(C)** Heat−map of the top 100 GEO train DEGs. **(D)** Volcano plot of pCR versus RD samples in the GEO training cohort (51 up− and 50 down−regulated DEGs).

In the TCGA cohort, a total of 984 DEGs were identified between tumor and normal tissues, including 678 downregulated and 306 upregulated genes (|log_2_FC| > 1, FDR < 0.05) (**Figures 2A, B**). Compared with normal tissues, the top three downregulated genes in the tumor tissues were FABP4, SAA1, and SFRP1, while the top three upregulated genes were COMP, COL10A1, and MMP11. In the GEO training cohort, differential expression analysis between the pCR and RD subgroups yielded 100 DEGs, comprising 49 downregulated and 51 upregulated genes (**Figures 2C, D**). Compared with RD patients, the top three downregulated genes in the pCR group were CEACAM6, ECM1, and AGR2, while the top three upregulated genes were CDC20, CCL8, and CCL18.

### 3.3 Feature gene selection via machine learning

**Figure 3:**
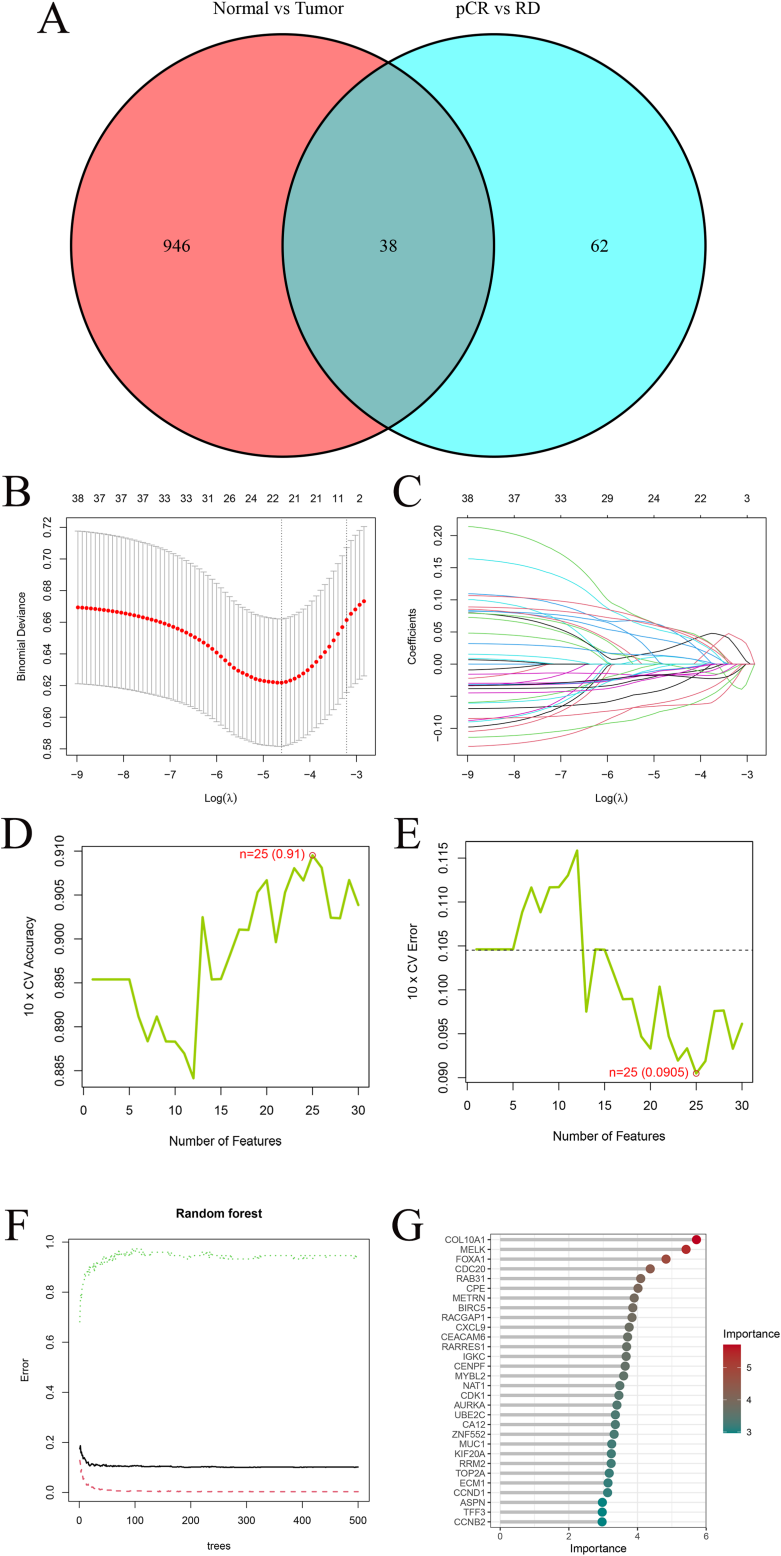
Machine−learning feature selection. **(A)** Venn diagram showing 38 overlapping DEGs between TCGA and GEO cohort analyses. **(B)** Ten-fold cross-validation to select the optimal λ in LASSO regression. The minimum binomial deviance was used to determine the best model. **(C)** LASSO coefficient profiles of the 38 candidate genes. Most coefficients shrink to zero as λ increases. **(D)** Ten-fold cross-validation accuracy curve for SVM-RFE. The highest accuracy (0.91) was achieved when 25 features were selected. **(E)** Ten-fold cross-validation error curve for SVM-RFE. The lowest error (0.0905) occurred with 25 features, indicating the optimal subset. **(F)** Random−forest variable−importance plot (MeanDecreaseGini); dashed line marks the cutoff. **(G)** Top 30 RF genes ordered by importance.

A total of 38 overlapping DEGs between the TCGA cohort and GEO training cohort comparisons were retained for subsequent machine learning-based feature selection (**Figure 3A**) (The full list of the 38 overlapping DEGs is provided in **Supplementary Table S1**). Among the 38 candidate genes, three machine learning algorithms were employed for feature selection. LASSO regression identified 22 genes with non-zero coefficients (**Figures 3B, C**). SVM-RFE yielded 25 genes with minimal cross-validation error (**Figures 3D, E**), while RF analysis retained 12 genes with a Mean Decrease Gini > 3.5 (**Figures 3F, G**), including COL10A1, MELK, FOXA1, CDC20, RAB31, CPE, METRN, BIRC5, RACGAP1, CXCL9, CEACAM6, and RARRES1.

### 3.4 Model construction and performance evaluation

**Figure 4:**
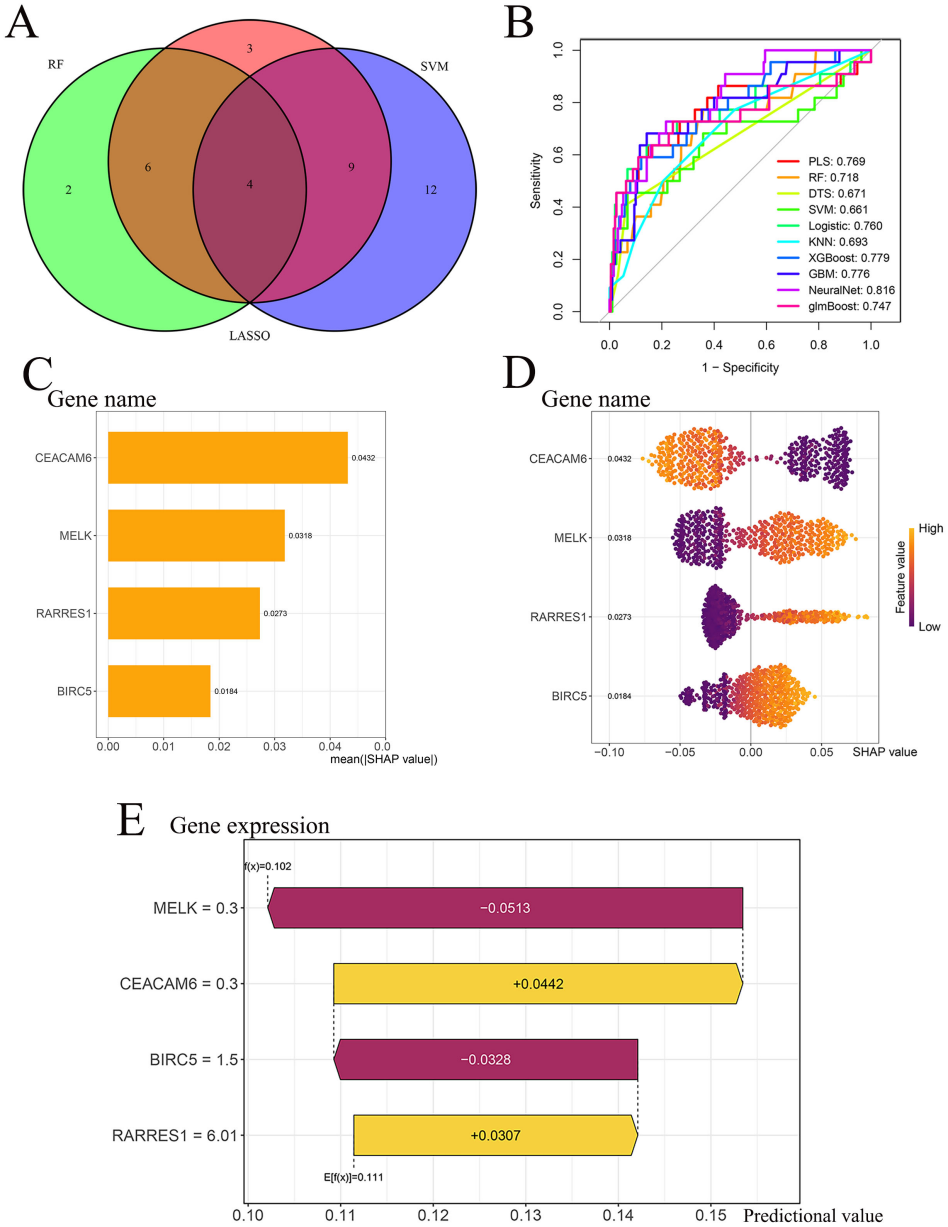
Model performance and SHAP interpretability. **(A)** Intersection of LASSO, RF and SVM−RFE outputs identifies four final feature genes (CEACAM6, MELK, RARRES1, BIRC5). **(B)** ROC curves of ten classifiers in the test cohort; the NeuralNet model achieves the highest AUC = 0.816. **(C)** SHAP summary bar plot for the NeuralNet model, ranked by mean absolute SHAP value. **(D)** SHAP bee−swarm plot illustrating gene impact direction and magnitude across individual patients. **(E)** SHAP waterfall plot for a representative patient illustrating gene−specific contributions to the NeuralNet prediction threshold.

Four genes-MELK, BIRC5, RARRES1, and CEACAM6-overlapped across LASSO, RF, and SVM-RFE analyses and were defined as the final feature genes for subsequent modeling (**Figure 4A**). Using the expression profiles of the final feature genes, ten machine learning models were developed based on the training cohort and tested on the independent test cohort. Among the ten algorithms, the NeuralNet demonstrated optimal performance in the test cohort (AUC = 0.816) (**Figure 4B**). The SHAP summary bar plot revealed that CEACAM6 contributed most significantly to the NeuralNet model’s predictive output, whereas BIRC5 had the smallest impact (**Figure 4C**). The SHAP bee−swarm plot (**Figure 4D**) illustrates the impact of each gene on individual predictions within the NeuralNet model. Higher expression of CEACAM6 was associated with negative SHAP values, indicating that increased CEACAM6 levels shifted the model’s prediction toward RD patient, thereby serving as a negative predictor for pCR response. In contrast, higher expression of MELK, RARRES1, and BIRC5 was associated with positive SHAP values, suggesting that these genes positively influenced the model’s prediction toward pCR status. These patterns highlight the opposing roles of CEACAM6 and the other three genes in the model’s discrimination of neoadjuvant chemotherapy response. The SHAP waterfall plot further elucidates gene−specific effects for a representative sample (**Figure 4E**). The model’s baseline expectation was E[f(x)] = 0.111. As an illustrative example, for a representative sample, MELK (expression = 0.30) decreased the predicted probability by 0.0513, BIRC5 (expression = 1.50) further decreased it by 0.0328, CEACAM6 (expression = 0.30) increased the probability by 0.0442, and RARRES1 (expression = 6.01) contributed an additional increase of 0.0307. These combined effects yielded a final prediction of f(x) = 0.102. Since the predicted value (f(x) = 0.102) was below the pCR classification threshold of 0.111, the sample was classified as residual disease (RD).

### 3.5 Expression patterns and prognostic value of feature genes

**Figure 5:**
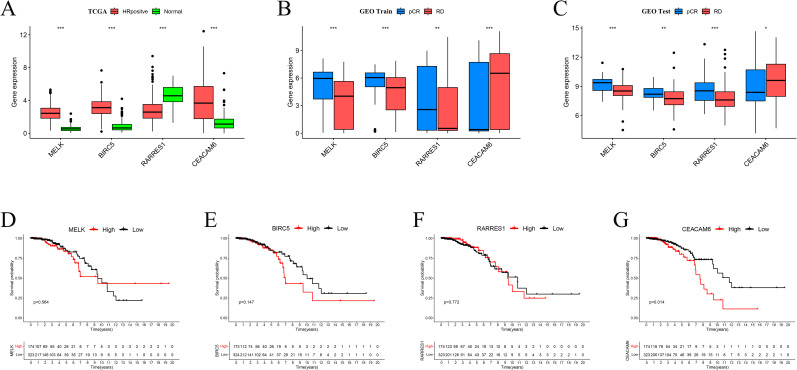
Expression patterns and prognostic impact of feature genes. **(A)** Boxplots comparing gene expression between TCGA normal and HR+/HER2-tumour tissues. **(B)** Expression differences between pCR and RD groups in the GEO training set. **(C)** Validation of expression trends in the GEO test set. Significance levels: *P < 0.05, **P < 0.01, ***P < 0.001. **(D-F)** Kaplan-Meier overall−survival curves for MELK, RARRES1 and BIRC5 (no significant difference). **(G)** High CEACAM6 expression correlates with poorer OS in HR+/HER2- breast cancer (log−rank p = 0.014).

Boxplot analyses revealed that MELK, BIRC5, and CEACAM6 were significantly overexpressed in HR+/HER2– breast cancer tissues compared to normal breast tissues in the TCGA cohort, whereas RARRES1 expression was markedly reduced in tumor tissues (p < 0.001; **Figure 5A**). In the GEO training cohort, MELK, BIRC5, and RARRES1 were significantly upregulated in pCR patients compared to RD patients, whereas CEACAM6 expression was markedly elevated in RD patients (p < 0.01; **Figure 5B**). Similar expression trends were validated in the GEO test cohort, with MELK, BIRC5, and RARRES1 showing higher expression in the pCR group and CEACAM6 remaining significantly higher in the RD group (**Figure 5C**). Kaplan-Meier survival analysis demonstrated that high CEACAM6 expression was significantly associated with worse overall survival in HR+/HER2– breast cancer patients (log-rank p = 0.014; **Figure 4G**), whereas no statistically significant association was observed for MELK, BIRC5, or RARRES1 (**Figures 5D–F**). Therefore, CEACAM6 was selected as the key gene for subsequent analysis.

### 3.6 Functional enrichment analysis of CEACAM6

**Figure 6:**
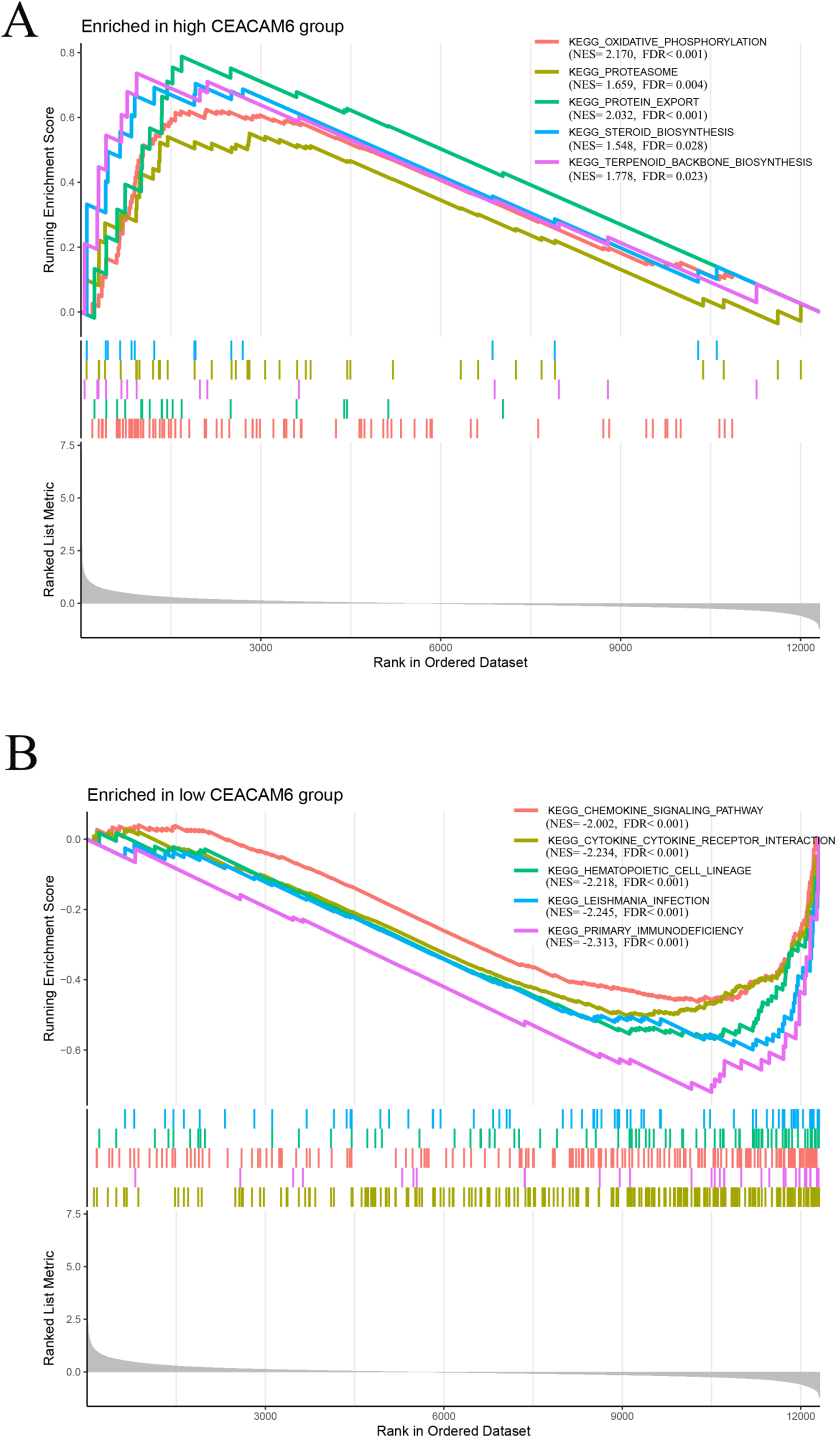
Gene−set enrichment analysis (GSEA) for CEACAM6. **(A)** Top five KEGG pathways enriched in the CEACAM6−high group (metabolism−related). **(B)** Top five pathways enriched in the CEACAM6−low group (immune−related). NES, normalised enrichment score; FDR < 0.05.

Gene Set Enrichment Analysis (GSEA) based on GEO train data revealed that high CEACAM6 expression was significantly associated with metabolism-related pathways, including oxidative phosphorylation, proteasome, protein export, steroid biosynthesis, and terpenoid backbone biosynthesis (**Figure 6A**). In contrast, low CEACAM6 expression was enriched in immune-related pathways such as chemokine signaling pathway, cytokine-cytokine receptor interaction, hematopoietic cell lineage, leishmania infection, and primary immunodeficiency (**Figure 6B**). These findings indicate that CEACAM6 overexpression may reflect a metabolically active but immunologically suppressed tumor phenotype, whereas lower CEACAM6 levels correlate with immune activation.

### 3.7 Immune cell landscape in pCR and RD groups and its correlation with CEACAM6

**Figure 7:**
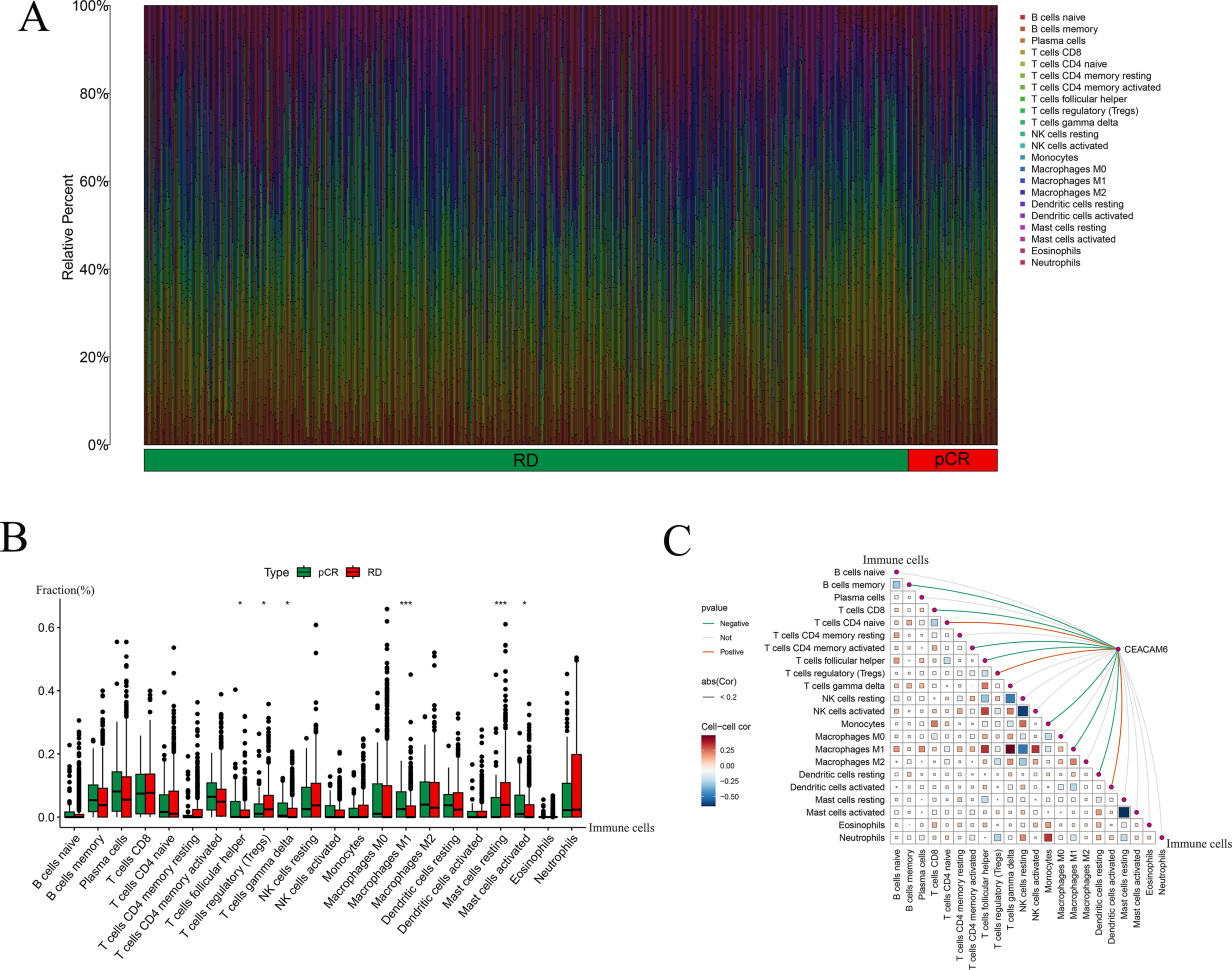
Immune landscape and correlation with CEACAM6 and pCR and RD samples. **(A)** Stacked bar chart of CIBERSORT−inferred immune−cell fractions for pCR and RD samples. **(B)** Boxplots showing differential infiltration between pCR and RD groups; Significance levels: *P < 0.05, ***P < 0.001. **(C)** Coupled correlation map depicting Spearman correlations between CEACAM6 expression and 22 immune−cell subsets; edge width reflects p, colour indicates direction and significance.

CIBERSORT analysis was used to evaluate the relative proportions of immune cell types in pCR and RD samples, including B cells, T cells, NK cells, macrophages, and dendritic cells, with samples categorized into pCR and RD groups (**Figure 7A**). CIBERSORT was applied to estimate the relative proportions of 22 immune cell types from bulk gene expression data, allowing for a detailed characterization of immune infiltration patterns between pCR and RD samples. Comparative analysis revealed that the pCR group exhibited significantly higher proportions of T cells follicular helper, T cells gamma delta, Macrophages M1, and Mast cells activated, while the fractions of T cells regulatory (Tregs) and Mast cells resting were significantly lower compared to the RD group (**Figure 7B**). These findings suggest that an immunologically active microenvironment characterized by enhanced effector cell infiltration and reduced immunosuppressive components may be associated with improved response to neoadjuvant chemotherapy. Spearman correlation analysis was performed to investigate the relationship between CEACAM6 expression and immune−cell infiltration levels. The results showed that CEACAM6 expression was positively correlated with T cells CD4 naive, T cells regulatory (Tregs), and Dendritic cells activated, whereas it was negatively correlated with B cells memory, T cells CD8, T cells CD4 memory activated, T cells follicular helper, Monocytes, Macrophages M1, and Dendritic cells resting (**Figure 7C**).

### 3.8 Drug sensitivity analysis

**Figure 8:**
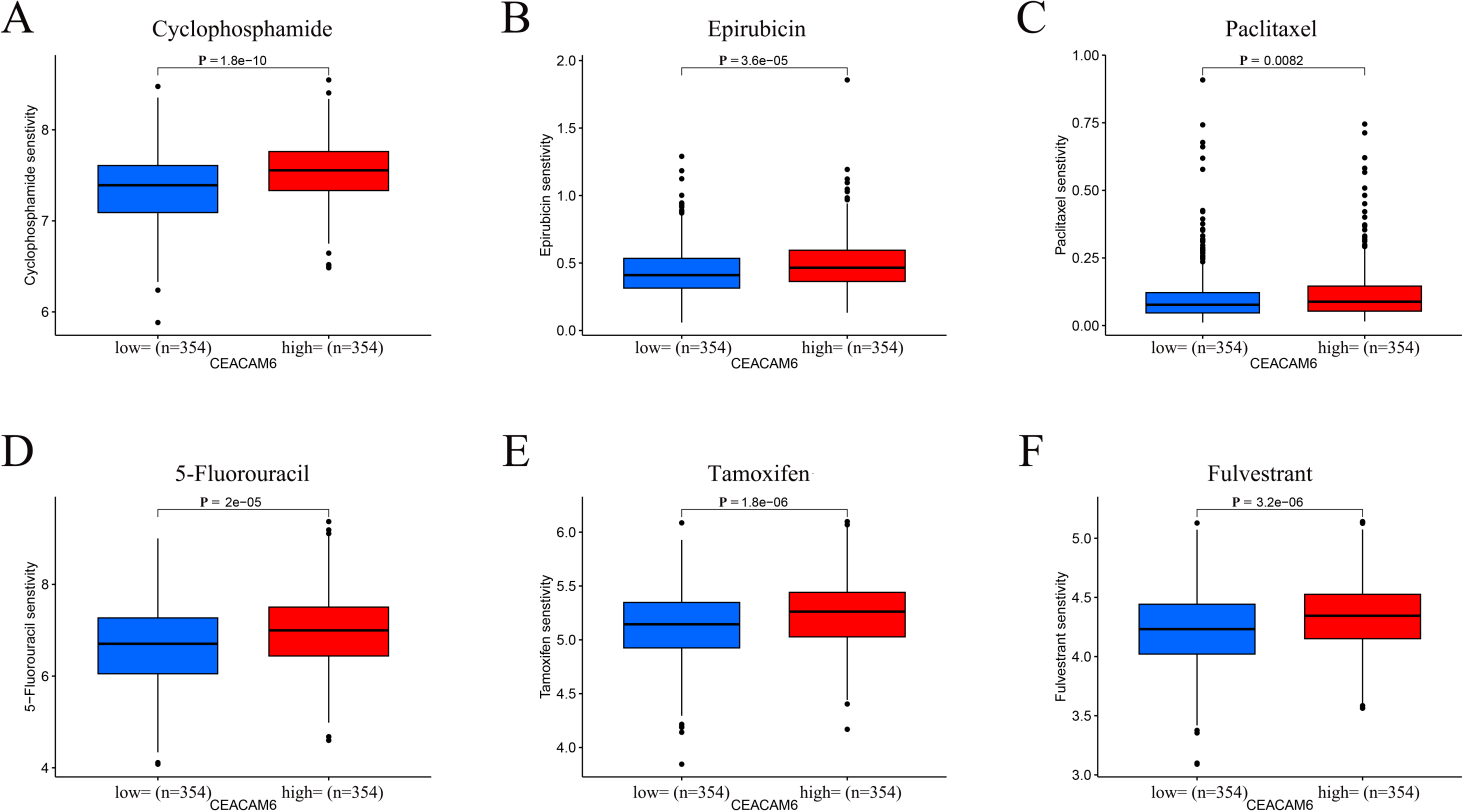
Predicted drug sensitivity stratified by CEACAM6 expression. **(A-F)** Boxplots of log_2_−transformed IC_50_ values for Cyclophosphamide, Epirubicin, Paclitaxel, 5−Fluorouracil, Tamoxifen and Fulvestrant in CEACAM6−high versus CEACAM6−low groups.

Drug sensitivity analysis using the oncoPredict package revealed that high expression of CEACAM6 was associated with increased predicted IC50 values for several commonly used chemotherapeutic and endocrine agents in HR+/HER2− breast cancer. These drugs included Cyclophosphamide, Epirubicin, Paclitaxel, 5-Fluorouracil, Tamoxifen, and Fulvestrant, suggesting a potential role of CEACAM6 in mediating drug resistance (**Figures 8A–F**).

### 3.9 CEACAM6 expression patterns in HR+/HER2- breast cancer tissues before and after NAC

**Figure 9:**
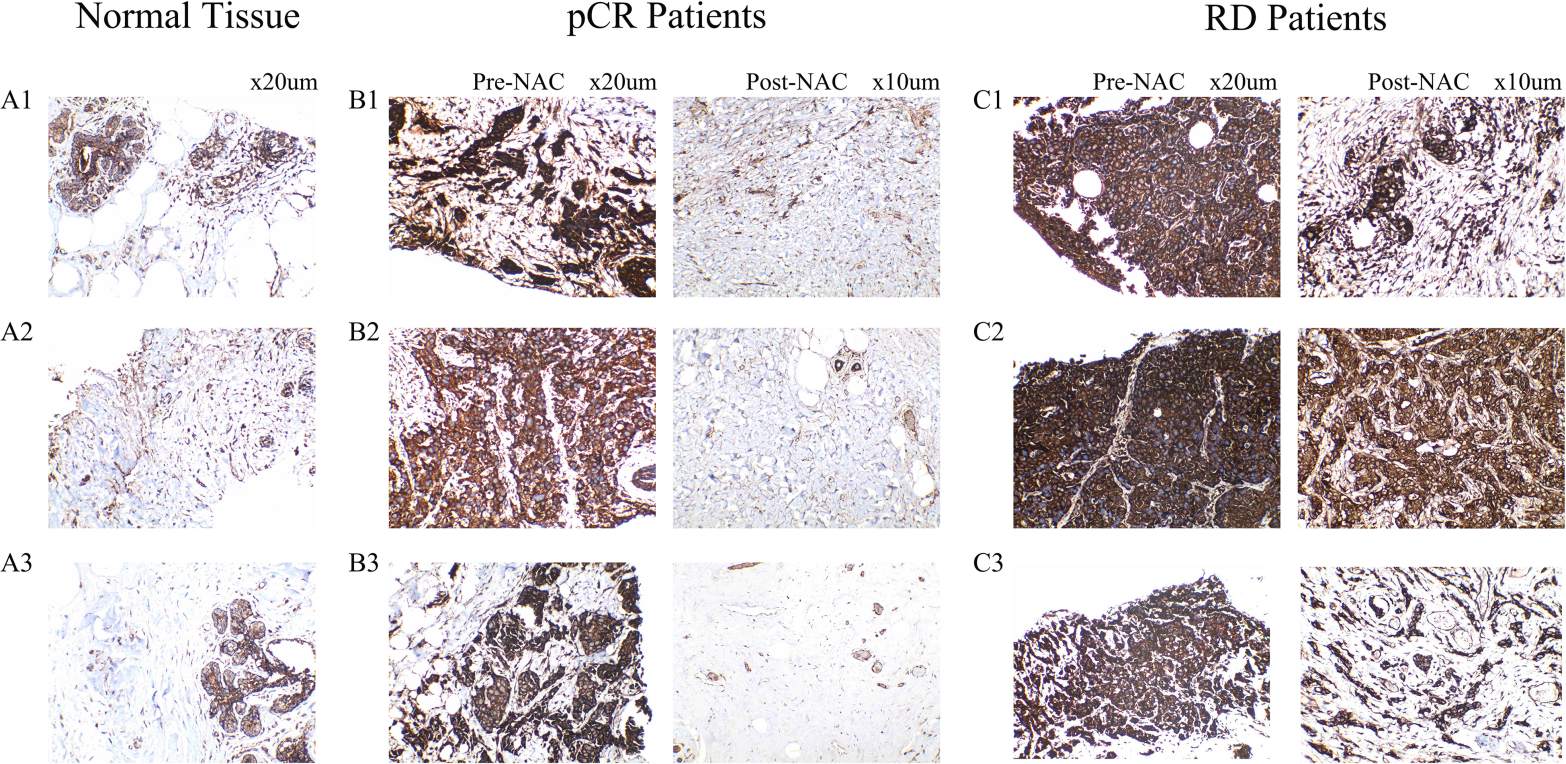
Representative immunohistochemical staining of CEACAM6 in breast tissue samples. **(A1-A3)** CEACAM6 expression in adjacent non-tumor tissues from three HR+/HER2- breast cancer patients without neoadjuvant chemotherapy. **(B1-B3)** CEACAM6 expression in tumor tissues from three patients who achieved pathological complete response (pCR), showing paired samples before (Pre-NAC) and after (Post-NAC) neoadjuvant chemotherapy. **(C1-C3)** CEACAM6 expression in tumor tissues from three patients with residual disease (RD), showing paired samples before (Pre-NAC) and after (Post-NAC) neoadjuvant chemotherapy.

Panels A1–A3 represent adjacent normal breast tissues (n = 3), while panels B1–B3 and C1–C3 correspond to tumor tissues from the pCR and RD groups (n = 3 each), respectively. Each case in the pCR and RD groups includes paired samples from the same patient before (Pre-) and after (Post-) NAC. In adjacent normal tissues (**Figures 9A1–A3**), CEACAM6 was weakly expressed, showing light brown membranous staining. In the pCR group, CEACAM6 expression was elevated before chemotherapy (Pre-NAC, **Figures 9B1–B3**), displaying strong and dense dark brown membranous staining. However, after chemotherapy (Post-NAC, **Figures 9B1–B3**), CEACAM6 expression was markedly reduced, either negative or limited to background levels, and even lower than that observed in normal tissues. In the RD group, CEACAM6 was strongly expressed both before (Pre-NAC, **Figures 9C1–C3**) and after chemotherapy (Post-NAC, **Figures 9C1–C3**), with persistent dark brown membranous staining, and no significant decrease following treatment. Notably, the Pre-NAC staining intensity of CEACAM6 in the RD group was stronger, more uniformly distributed, and denser compared to the pCR group. These findings suggest that CEACAM6 expression is downregulated only in patients who achieve pathological complete response (pCR), while remaining elevated in those with residual disease (RD). This indicates that relatively lower CEACAM6 expression levels before chemotherapy are associated with achieving pCR following NAC, supporting its potential as both a predictive and therapeutic biomarker for the efficacy of neoadjuvant treatment in breast cancer.

### 3.10 Comparison of clinical and hematological characteristics between pCR and RD HR+/HER2- breast cancer patients

Patients achieving pCR (n = 10) and those with RD (n = 96) showed no significant differences in age (49.2 ± 10.7 *vs*. 48.7 ± 9.7 years, P = 0.900), BMI (23.85 ± 3.93 *vs*. 24.44 ± 3.54, P = 0.621), or neutrophil count (3.94 ± 1.11 *vs*. 3.89 ± 1.12 × 10^9^/L, P = 0.905). However, lymphocyte count was significantly higher in the pCR group compared with the RD group (2.58 ± 0.45 *vs*. 2.09 ± 0.56 × 10^9^/L, P = 0.008), while NLR showed no significant difference (1.8 ± 0.6 *vs*. 1.9 ± 0.8, P = 0.593). Menopausal status, ER and PR expression, Ki-67 index, TNM stage, and histological grade also did not differ significantly between groups (P > 0.05), except for lymph node status, where the pCR group had a higher proportion of N2-3 (P = 0.004). Metastasis status showed a trend toward significance (P = 0.126) **Table 2**.

**Table 2:** Baseline characteristics of 106 NAC patients with HR+/HER2- breast cancer.

Parameter		pCR (n=10)	RD (n=96)	t/X^2^	P
Age	X±SD	49.20 ± 10.67	48.78 ± 9.97	0.126	0.900
BMI	X±SD	23.85 ± 3.93	24.44 ± 3.54	0.496	0.621
Neutrophil count (× 10^9^/L)	X±SD	3.94 ± 1.11	3.89 ± 1.14	0.120	0.905
Lymphocyte count (× 10^9^/L)	X±SD	2.58 ± 0.45	2.09 ± 0.56	2.725	0.008
NLR	X±SD	1.80 ± 0.61	1.93 ± 0.75	0.537	0.593
Menopausal Status	Premenopausal	5	57	–	0.738
	Postmenopausal	5	39		
Estrogen Receptor	<80%	3	31	–	1.000*
	>=80%	7	65		
Progesterone Receptor	<80%	8	69	–	0.724*
	>=80%	2	27		
Ki-67 Index	<30%	6	53	–	1.000*
	>=30%	4	43		
Tumor size	T1-2	5	50	–	1.000*
	T3-4	5	46		
Lymph Node status	N0-1	4	81	–	0.004*
	N2-3	6	15		
Metastasis status	M0	7	85		0.126*
	M1	3	11		
Histological Grade	G1	4	28	–	0.740*
	G2	3	39		
	G3	3	29		

* P-values were derived from Fisher’s exact test due to expected cell frequencies < 5, ensuring robust statistical inference; NLR, Neutrophil-to-lymphocyte ratio; X±SD, Mean ± Standard Deviation; BMI, Body mass index; pCR, pathological complete response; RD, Residual disease.

## 4 Discussion

This study aimed to identify molecular prognostic biomarkers associated with neoadjuvant chemotherapy (NAC) response and the immune microenvironment in hormone receptor-positive (HR+)/HER2-negative breast cancer. By integrating transcriptomic data from TCGA and multiple GEO datasets, we identified differentially expressed genes (DEGs) and employed three machine learning algorithms-LASSO, Random Forest, and SVM-RFE-to screen for four robust predictive genes: CEACAM6, MELK, RARRES1, and BIRC5. Using these genes, we constructed various machine learning models, which demonstrated favorable performance in independent test cohorts. CEACAM6 emerged as a key gene significantly associated with overall survival in HR+/HER2- breast cancer. Its expression was validated at both the mRNA and protein levels in normal tissue and paired tumor specimens before and after NAC. Immune infiltration analysis revealed distinct immune landscapes between patients achieving pathological complete response (pCR) and those with residual disease (RD), and CEACAM6 expression correlated significantly with multiple immune cell subsets. A retrospective review of clinical data from 106 HR+/HER2- breast cancer patients receiving NAC further indicated that baseline total lymphocyte count and nodal status were significantly associated with NAC sensitivity. GSEA and drug sensitivity analyses demonstrated that CEACAM6 high-expression tumors exhibited upregulation of multiple chemoresistance-related pathways and higher IC50 values for both chemotherapy and endocrine therapy agents, suggesting a role for CEACAM6 in mediating drug resistance.

In total, we included 497 HR+/HER2- breast cancer samples from TCGA for DEG analysis and integrated nine GEO datasets (n = 956) divided into two cohorts for biomarker identification between pCR and RD groups. Compared to prior studies, which often relied on a single public dataset and limited analytical approaches, our study provides a more comprehensive framework by combining large-scale data integration, robust feature selection through three distinct algorithms, and extensive external validation (25, 26). While prior studies have used LASSO or Random Forest to predict NAC outcomes (16, 17), these efforts often lacked cross-validation and clinical applicability. Moreover, few models have focused specifically on the HR+/HER2- subtype or integrated immune context into NAC response prediction. Given the relatively low pCR rate in HR+/HER2- breast cancer (10-15%, consistent with our datasets) (3), our cohort offers valuable clinical insight. By combining three independent machine learning methods (LASSO, SVM-RFE, Random Forest), and evaluating their generalizability using multiple models including neural networks, our approach showed consistently high AUCs in large merged test cohorts. We further applied SHAP for model interpretability. Notably, we conducted immunohistochemical analysis using paired samples from nine patients, confirming that CEACAM6 was upregulated in tumors compared to adjacent normal tissue, and that pCR patients exhibited lower CEACAM6 levels than RD patients. Moreover, CEACAM6 remained elevated in RD tumors before and after NAC, supporting its role as a predictive biomarker. Among the four model-selected genes, CEACAM6 emerged as the foremost predictor, with robust statistical and biological significance. It was significantly associated with poor overall survival and remained highly expressed in chemoresistant tumors. CEACAM6 encodes a GPI-anchored membrane protein involved in cell adhesion, invasion, and immune evasion, and has been linked to activation of PI3K/AKT and EMT pathways (27). GSEA revealed enrichment of oxidative phosphorylation and proteasome pathways in CEACAM6-high tumors, supporting its role in chemoresistance. Immune profiling showed that CEACAM6 correlated with higher Treg infiltration and lower CD8+ T cell levels, indicating a more immunosuppressive microenvironment. While BIRC5, MELK, and RARRES1 may complement CEACAM6’s function, its multifaceted involvement in metabolic reprogramming, immune suppression, and survival pathways may explain its leading role in our model. Our clinical data analysis (n = 106) revealed that pCR patients had higher pre-treatment absolute lymphocyte counts, and patients with N2–N3 nodal status were more likely to achieve pCR. This highlights the potential role of host immune status in NAC response. Previous studies have proposed peripheral lymphocyte count as a potential predictor of NAC efficacy. A Korean retrospective study found that higher baseline and on-treatment lymphocyte counts correlated with pCR in breast cancer (11), consistent with our findings. Additionally, multiple studies have shown that higher tumor-infiltrating lymphocytes (TILs), particularly CD8+ T cells, are associated with pCR across breast cancer subtypes (9, 10, 28). While most previous work focused on TNBC or HER2-positive tumors, our findings newly demonstrate this trend within HR+/HER2- tumors. Interestingly, our observation that patients with N2–N3 nodal involvement showed higher pCR rates may seem paradoxical. However, this has been hypothesized to result from increased antigen exposure driving local immune activation and heightened chemotherapy sensitivity (29). This supports the concept that regional immune activation may facilitate better treatment responses. However, given the retrospective nature of this analysis and the potential for selection bias, these findings should be interpreted cautiously and require validation in future prospective studies.

CEACAM6 (Carcinoembryonic Antigen-Related Cell Adhesion Molecule 6) encodes a glycosylphosphatidylinositol (GPI)-anchored cell surface glycoprotein belonging to the carcinoembryonic antigen (CEA) family. It has been implicated in cell adhesion, promotion of tumor cell proliferation and invasion, as well as in mediating resistance to chemotherapy (27, 30). In this study, we found that patients with high CEACAM6 expression were more likely to have residual disease (RD) after neoadjuvant chemotherapy, whereas those with low expression were more likely to achieve pathological complete response (pCR). Moreover, high CEACAM6 expression was also associated with worse prognosis, suggesting that its upregulation may impair the efficacy of systemic therapies, including chemotherapy and endocrine therapy, thereby leading to poor clinical outcomes. Our results align with Burgos et al.’s meta-analysis linking CEACAM6 overexpression was associated with adverse prognosis in several malignancies, including breast, gastric, and colorectal cancers (31). Beyond transcriptomic profiling, we further explored the relationship between CEACAM6 expression and drug sensitivity using the oncoPredict algorithm trained on the GDSC2 database. The results showed that HR+/HER2− breast cancer samples with high CEACAM6 expression had significantly elevated predicted IC50 values for several commonly used chemotherapeutic agents, including cyclophosphamide, epirubicin, paclitaxel, and 5-fluorouracil, as well as for endocrine therapies such as tamoxifen and fulvestrant. These findings suggest that CEACAM6-high tumors may be intrinsically less responsive to both chemotherapy and endocrine therapy. However, as oncoPredict relies on cell line–based training data, it does not fully capture the hormonal and immune features of clinical HR+/HER2− tumors, limiting its direct clinical translation. The drug sensitivity results based on oncoPredict are predictive in nature and should be interpreted cautiously, particularly given the limited number of HR+/HER2− cell lines in the GDSC2 dataset. Further validation using cell-based assays and preclinical models specific to this subtype is necessary to confirm the functional role of CEACAM6 in mediating chemoresistance. To support the predictive findings, we performed immunohistochemical (IHC) analysis in an independent clinical cohort. CEACAM6 expression was significantly higher in patients with poor response to neoadjuvant chemotherapy, providing additional evidence for its potential role in treatment resistance. Collectively, these data suggest that CEACAM6 may serve as a marker of reduced therapeutic sensitivity, whereas low CEACAM6 expression may identify patients more likely to benefit from standard treatment regimens.

To further elucidate the potential mechanisms underlying CEACAM6-mediated drug resistance, we performed GSEA between high and low CEACAM6 expression groups. The results showed that tumors with high CEACAM6 expression were significantly enriched in pathways related to oxidative phosphorylation, proteasome function, protein export, steroid biosynthesis, and terpenoid backbone biosynthesis—pathways that have been widely linked to chemotherapy resistance. Enhanced oxidative phosphorylation, for example, is a hallmark of metabolic reprogramming in cancer cells, promoting ATP production and reactive oxygen species (ROS) clearance, which together contribute to resistance against chemotherapeutic agents (32). The proteasome pathway, when activated, facilitates the degradation of cyclins and apoptosis-regulating proteins, promoting tumor cell survival and evasion of chemotherapy-induced cell death (33). Aberrant proteasome activity has been linked to resistance in various solid tumors, and pharmacologic inhibition of this pathway has been shown to restore chemosensitivity (34, 35). Protein export is another key mechanism maintaining tumor homeostasis and intercellular communication. This pathway is responsible for exporting newly synthesized proteins from ribosomes to the extracellular space or specific organelles. Its upregulation has been implicated in the enhanced transport and expression of anti-apoptotic and drug resistance-associated molecules (36, 37). In addition, enrichment of steroid biosynthesis and terpenoid backbone biosynthesis pathways is notable. Steroid-derived metabolites can stabilize cell membranes and activate lipid signaling pathways, contributing to tumor cell survival and chemoresistance. Previous studies have shown that upregulation of these pathways can reduce tumor sensitivity to taxanes and anthracyclines (38). Terpenoid biosynthesis, upstream of steroid synthesis, plays a crucial role in drug metabolism and membrane transport, influencing drug uptake and efflux (39). Conversely, tumors with low CEACAM6 expression were significantly enriched in immune-related pathways, including the chemokine signaling pathway and cytokine–cytokine receptor interaction pathway. This suggests that low CEACAM6 expression may facilitate immune activation, thereby enhance antitumor immunity and improve responsiveness to neoadjuvant chemotherapy. Specifically, the chemokine signaling pathway plays a pivotal role in recruiting immune cells and shaping the immune microenvironment. Upregulation of chemokines can attract CD8^+^ T cells, NK cells, and dendritic cells into the tumor tissue, thereby enhancing local immune activity and promoting tumor cell apoptosis in response to chemotherapy (40). In parallel, the cytokine–cytokine receptor interaction pathway promotes Th1 immune responses and inflammatory cytokine secretion, which are known to synergize with chemotherapy-induced immunogenic tumor cell death (41). Mechanistically, oxidative phosphorylation supports chemoresistance by supplying ATP for survival processes, enhancing mitochondrial fitness, and reducing ROS-induced apoptosis (42). This metabolic adaptation may allow CEACAM6-high tumor cells to better withstand the metabolic stress imposed by cytotoxic chemotherapy. Additionally, enrichment of proteasome-related pathways may promote the clearance of misfolded or damaged proteins, contributing to resistance by maintaining proteostasis and suppressing apoptosis (43). Conversely, the enrichment of immune-related pathways in CEACAM6-low tumors, particularly those involving chemokine signaling and antigen presentation, suggests a more immune-permissive tumor microenvironment (44). This observation is consistent with our CIBERSORT analysis, where CEACAM6-low tumors exhibited higher infiltration of effector immune cells, potentially enhancing antitumor immunity and chemosensitivity. Taken together, these findings support a model in which high CEACAM6 expression activates multiple chemoresistance-associated pathways—including metabolic and protein degradation networks—while low CEACAM6 expression is associated with immune pathway activation and enhanced antitumor immunity. These observations further substantiate CEACAM6 as a promising predictive biomarker for chemotherapy sensitivity in HR+/HER2- breast cancer. Importantly, we acknowledge that the immune mechanisms and drug resistance pathways identified in this study are primarily derived from computational analyses and correlation-based models. While our findings demonstrate strong associations between CEACAM6 expression, immune infiltration patterns, and predicted drug responses, these results do not establish direct causality. Therefore, they should be interpreted with caution. Further experimental validation through *in vitro* and *in vivo* studies is needed to elucidate the mechanistic role of CEACAM6 in mediating immune modulation and chemoresistance. These results are hypothesis-generating and provide a framework for future mechanistic exploration. Additionally, although subgroup analysis based on TIL levels could offer deeper insight into CEACAM6-mediated immune suppression, such analysis was not feasible due to the lack of standardized TIL annotations in the available datasets. Future studies incorporating pathological or digital TIL quantification may help determine whether the immunosuppressive effects of CEACAM6 are more pronounced in TIL-high tumors.

Further immune deconvolution via CIBERSORT revealed that pCR tumors were enriched with effector immune cells (Tfh, γδ T cells, M1 macrophages, activated NK cells), while RD tumors harbored higher proportions of immunosuppressive cells (Tregs, resting mast cells, naive CD4+ T cells). This suggests that chemosensitive tumors possess pre-existing immune-active microenvironments, whereas chemoresistant tumors exhibit immune evasion features. Regression analysis showed CEACAM6 positively correlated with suppressive cells (Tregs, naive CD4+ T cells, activated dendritic cells) and negatively with effector populations (memory B cells, activated CD4+ T cells, CD8+ T cells, Tfh, M1 macrophages). These observations align with prior findings that CEACAM6 may mediate immunosuppression. Pinkert et al. demonstrated that CEACAM6 suppresses T cell activation via CEACAM1 interaction, and that blocking this axis with the humanized antibody BAY1834942 restores cytokine secretion and cytotoxicity (45). Nicolas et al. further showed that Tregs in breast tumors contribute to immune suppression and tumor progression (46). Other studies confirmed that Tregs inhibit effector T cells through cytokines like TGF-β and IL-10 (47, 48). In lung adenocarcinoma, CEACAM6 was shown to enhance EMT and stemness, contributing to cisplatin resistance and immune escape via suppression of immune pathways (49). In breast cancer, CEACAM6 has been linked to paclitaxel resistance through PI3K/AKT pathway activation and apoptosis inhibition (27). Maraqa et al. previously reported higher CEACAM6 expression in tamoxifen-resistant breast tumors (50), implicating it in endocrine resistance. Recent preclinical studies have explored CEACAM6 as a therapeutic target. Nakazawa et al. developed an antibody-drug conjugate (84-EBET) targeting CEACAM6, which delivered degradation agents to pancreatic tumors and significantly enhanced the efficacy of chemotherapy and immunotherapy (51). Compared to well-established biomarkers in HR+/HER2− breast cancer, such as ESR1 mutations and PI3K/AKT pathway genes, CEACAM6 may offer complementary predictive value by capturing both chemotherapy sensitivity and immune microenvironment status. While ESR1 mutations primarily predict endocrine resistance (52), and PI3K/AKT activation is associated with survival signaling and drug resistance, CEACAM6 appears to integrate both survival and immune regulatory functions. Prior studies in gastric and pancreatic cancers have shown that CEACAM6 activates PI3K/AKT signaling (52, 53), supporting the mechanistic overlap observed in our study. Although we did not directly evaluate ESR1 or PI3K/AKT gene alterations, incorporating CEACAM6 into multi-marker predictive models may enhance patient stratification and inform more personalized therapeutic approaches in HR+/HER2− breast cancer. These findings support CEACAM6 as a dual regulator of drug response and immune evasion. The immunosuppressive profile of CEACAM6-high tumors—characterized by increased Tregs and naïve CD4+ T cells and reduced CD8+ T cells and M1 macrophages—suggests impaired antitumor immunity. Mechanistically, CEACAM6 may promote immune evasion through interaction with CEACAM1 and induction of TGF-β/IL-10 signaling, both of which inhibit effector T cell function. This immunosuppressive environment may partially explain the reduced pCR rates observed in CEACAM6-high patients. Targeting CEACAM6 with monoclonal antibodies has shown potential in reversing immune suppression in preclinical studies, supporting its relevance as a therapeutic target in HR+/HER2− breast cancer.

Despite encouraging findings, this study has limitations. In our external independent cohort, the number of patients who achieved pCR (n = 10) was relatively low, representing 9.43% of the total 106 HR+/HER2− breast cancer cases. This distribution reflects the real-world clinical situation, as HR+/HER2− tumors generally exhibit lower sensitivity to neoadjuvant chemotherapy and are thus less frequently selected for NAC. Only in recent years has our center begun implementing NAC protocols for this subtype in a more systematic manner. Notably, the observed pCR rate in our cohort is comparable to that of the GEO training (10.45%) and test (12.90%) cohorts, supporting the clinical relevance of our findings. Nevertheless, we acknowledge that the limited pCR sample size may introduce analytical bias, and we plan to conduct larger-scale prospective studies in the future to better evaluate chemotherapy sensitivity in this population. Immune infiltration was estimated computationally using CIBERSORT, requiring further experimental validation. Immunohistochemical analysis was limited by sample size. Specifically, only nine patients were included in the IHC analysis due to limited availability of high-quality, matched pre- and post-treatment samples. We recognize that this small sample size may lead to selection bias and reduce the generalizability of the results. In future studies, we plan to expand the IHC cohort through prospective, multi-center collaborations to strengthen the translational validity of CEACAM6 as a predictive biomarker. Our findings suggest that CEACAM6 may function not only as a predictor of chemotherapy response but also as a potential therapeutic target in HR+/HER2− breast cancer. Given its detectable expression via IHC or qPCR in biopsy samples, CEACAM6 could be integrated into clinical decision-making frameworks to identify patients less likely to benefit from standard neoadjuvant chemotherapy. Functional studies are needed to manipulate CEACAM6 expression *in vitro* and *in vivo*, and to evaluate CEACAM6-targeted agents such as BAY1834942 in preclinical models. In addition, single-cell sequencing and spatial immune profiling could help clarify how CEACAM6 influences the tumor immune microenvironment. Lastly, combining CEACAM6 expression with clinical parameters—such as tumor grade and receptor status—may enhance the predictive performance of future clinical models.

In conclusion, our study identifies CEACAM6 as a potential biomarker of NAC response in HR+/HER2- breast cancer and implicates it in mediating immune evasion. The interplay between tumor-intrinsic features and host immunity appears critical to chemotherapy sensitivity. Our data suggest that CEACAM6-high tumors tend toward immunosuppressive environments, while low-expression tumors display active immune responses conducive to pCR. Targeting CEACAM6, for instance with BAY1834942, may improve NAC outcomes and warrants clinical evaluation. Incorporating CEACAM6 expression into machine learning-based predictive models may also guide personalized therapeutic decisions. Our findings highlight the value of integrating computational and experimental approaches to refine treatment strategies for HR+/HER2- breast cancer.

## 5 Conclusion

This study identifies CEACAM6 as a robust predictive biomarker for neoadjuvant chemotherapy (NAC) response in hormone receptor-positive/human epidermal growth factor receptor 2-negative (HR+/HER2−) breast cancer. Through multi-cohort transcriptomic integration and machine learning–based feature selection, CEACAM6 was consistently associated with chemoresistance, poor survival, immunosuppressive infiltration, and reduced drug sensitivity. High CEACAM6 expression correlated with activation of metabolic and proteasome pathways, as well as increased regulatory T cell infiltration and diminished cytotoxic immune cell presence. In contrast, CEACAM6-low tumors exhibited immune activation and greater NAC responsiveness. Immunohistochemical validation and retrospective clinical analysis further reinforced its predictive and translational relevance. These findings underscore the potential of CEACAM6 not only as a prognostic biomarker but also as a therapeutic target. Integrating CEACAM6 into predictive frameworks alongside immune and pathway features may enhance individualized treatment strategies and improve clinical outcomes in HR+/HER2− breast cancer.

## Data Availability

The original contributions presented in the study are included in the article/**Supplementary Material**. Further inquiries can be directed to the corresponding authors.
